# Parts & Pools: A Framework for Modular Design of Synthetic Gene Circuits

**DOI:** 10.3389/fbioe.2014.00042

**Published:** 2014-10-06

**Authors:** Mario Andrea Marchisio

**Affiliations:** ^1^School of Life Science and Technology, Harbin Institute of Technology, Harbin, China

**Keywords:** Parts, Pools, gene circuits, Boolean gates, synthetic biology, modeling

## Abstract

Published in 2008, Parts & Pools represents one of the first attempts to conceptualize the modular design of bacterial synthetic gene circuits with Standard Biological Parts (DNA segments) and Pools of molecules referred to as common signal carriers (e.g., RNA polymerases and ribosomes). The original framework for modeling bacterial components and designing prokaryotic circuits evolved over the last years and brought, first, to the development of an algorithm for the automatic design of Boolean gene circuits. This is a remarkable achievement since gene digital circuits have a broad range of applications that goes from biosensors for health and environment care to computational devices. More recently, Parts & Pools was enabled to give a proper formal description of eukaryotic biological circuit components. This was possible by employing a rule-based modeling approach, a technique that permits a faithful calculation of all the species and reactions involved in complex systems such as eukaryotic cells and compartments. In this way, Parts & Pools is currently suitable for the visual and modular design of synthetic gene circuits in yeast and mammalian cells too.

## Introduction

**Figure 1:**
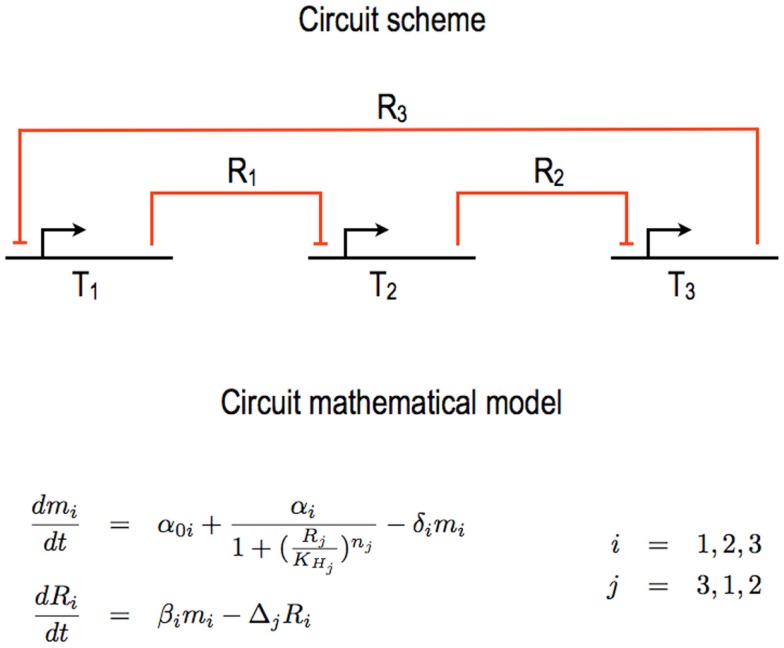
**Bacterial repressilator**. One of the first synthetic gene circuits realized in *E. coli* is the so called *repressilator* (Elowitz and Leibler, 2000). Its core scheme is organized in three transcription units (*T_i_, i* = 1, …, 3) wired together via the exchange of as many different repressor proteins (*R_i_, i* = 1, …, 3). The original circuit design and mathematical model (as re-proposed in this figure) neglect the transcription unit structure in DNA Parts and any specific interactions between, on one hand, RNA polymerases and the DNA and, on the other hand, ribosomes and the mRNA. Furthermore, the action of the repressors on their target promoters is lumped into Hill functions. The overall mathematical model requires six ODEs, two for each unit. Here, *α*_0_*_i_* represents the leakage rate constants, *α_i_* the transcription rate constants, *β_i_* the translation rate constants; *δ_i_* and Δ*_i_* are the mRNA and protein decay rate constants, respectively; $K_{H_{j}}$ are the Hill constants and *n_j_* the Hill (cooperativity) coefficients.

Early works in Synthetic Biology are small circuits engineered mainly in *E. coli* (Andrianantoandro et al., 2006). These works show how even simple mathematical models can drive wet-lab circuit implementation. Stability analysis permits to determine the range of some parameter values (e.g., protein degradation rates) in order to obtain specific behaviors such as sustained oscillations or switch between two stable states [see for instance (Atkinson et al., 2003)]. Basic modules for circuit design and modeling are transcription units. Wires are transcription factors (repressors or activators – see Figure 1). In an attempt to map concepts from electrical engineering into biology, Drew Endy suggests, in 2005, to take as components for bacterial circuits the Standard Biological Parts stored at the MIT Registry (Endy, 2005). There, they are organized into different categories: promoters, RBSs (Ribosome Binding Sites), coding regions for proteins and sRNAs (small RNAs), and terminators. Biological counterpart of the electrons are identified in the so called *common signal carriers* namely RNA polymerases and ribosomes. Their fluxes [Polymerases Per Second (PoPS), and Ribosomes Per Second (RiPS)] can be considered as biological currents that flow through all the Parts on the DNA (PoPS) and the mRNA (RiPS). They represent a *shared input/output* signal that permits Parts’ composition into biological devices such as transcription units or inverters.

According to these ideas, several computational tools for synthetic gene circuit modeling and design were developed in the past years (see Table 1). One of the first to be published is our method – and the corresponding software – that shows how to design gene circuits by connecting two different kinds of modules: Standard Biological Parts and Pools storing signal carrier molecules. We did not implement these concepts into an independent piece of software but instead our method is an add-on for the Process Modeling Tool (ProMoT) (Mirschel et al., 2009). In this way, for instance, a user can exploit the ProMoT Graphical User Interface (GUI) to design gene circuits in a drag & drop way, exactly like in electronics. We did not give any particular name to our collection of scripts that, later on, was just cited as ProMoT. However, what we presented in Marchisio and Stelling (2008) is a method of a broader scope, independent, in principle, of ProMoT, and any other available tool. Here, we will refer to our theoretical framework (and its *in silico* realization) as *Parts & Pools*.

**Table 1:** **Software for synthetic gene circuit design**.

Software	URL	Reference
BioJADE	http://web.mit.edu/jagoler/www/biojade/	Goler (2004)
AutoBioCAD	http://soft.synth-bio.org/amsparts.html	Rodrigo and Jaramillo (2013)
Parts & Pools	Available on request	Marchisio and Stelling (2008)
ProMoT	http://www.mpi-magdeburg.mpg.de/projects/promot/	Mirschel et al. (2009)
SynBioSS	http://synbioss.sourceforge.net/	Hill et al. (2008)
iBioSim	http://www.async.ece.utah.edu/iBioSim/	Myers et al. (2009)
TinkerCell	http://www.tinkercell.com/	Chandran et al. (2009)
GenoCAD	http://www.genocad.org	Cai et al. (2007) and Czar et al. (2009)
GEC	http://research.microsoft.com/en-us/projects/gec/	Pedersen and Phillips (2009)
VirtualParts	http://models.cellml.org/	Cooling et al. (2010)
Clotho	http://www.clothocad.org/	Xia et al. (2011)
TASBE	Multiple tools	Beal et al. (2012)
SBROME	http://tagkopouloslab.ucdavis.edu/software.html	Huynh et al. (2013)

*BioJADE is a precursor piece of software that shows how visual design principles can be applied to synthetic genetic circuits. Asmparts (Rodrigo et al., 2007a) – now included into AutoBioCAD – introduces the usage of fluxes such as PoPS and RiPS to connect DNA segments. Here, circuit components are SBML files assembled into a circuit from the command line. Parts & Pools provides more detailed bio-chemical models for DNA Parts based on full mass-action kinetics. Moreover, Parts & Pools extends the set of common signal carriers and assigns to them new circuit components, the Pools. Developed as an add-on of ProMoT, it allows designing gene circuits both in a drag & drop way and with a formal language, the Model Definition Language [MDL (Ginkel et al., 2003)]. All the other software for synthetic biology does not consider Pools as circuit components. SynBioSS and iBioSim supply the users with valuable analysis tools. The latter, in particular, allows modeling and simulations also at cell population level (Stevens and Myers, 2013). GenoCAD and GEC use grammars developed *ad hoc* to specify circuit structure and retrieve DNA Parts from dedicated databases. Connection to a DNA sequence repository, together with a visual editor, is offered also by TinkerCell. Virtual Parts is a repository of abstract part models encoded in CellML (Cuellar et al., 2003), whereas Clotho and TASBE represent workflows of computational tools from circuit design to wet-lab implementation. Finally, SBROME [as well as MatchMaker (Yaman et al., 2012), a component of the TASBE workflow, and iBioSim (Roehner and Myers, 2014)] translates an abstract circuit structure into an actual one where circuit components, retrieved from a library, are optimized in order to reduce circuit complexity (Huynh and Tagkopoulos, 2014). For a more detailed description and comparison of computational tools for Synthetic Biology we refer the reader to MacDonald et al. (2011), Marchisio (2012), Myers (2013), and Huynh and Tagkopoulos (2014)*.

This review paper is organized as follows: Section 1 presents Parts & Pools foundational ideas and its application to bacterial gene circuit design; Section 2 introduces the algorithm for the automatic design of gene digital circuits (the first in the field) that we developed starting from Parts & Pools; Section 3 shows how the rule-based modeling approach (Faeder et al., 2009) realized by the software BioNetGen (Blinov et al., 2004) was integrated into Parts & Pools in order to design eukaryotic modules and networks. In Section 4, finally, possible future extensions and improvements of Parts & Pools are discussed.

## Bacterial Circuits

### Design with Parts & Pools

**Figure 2:**
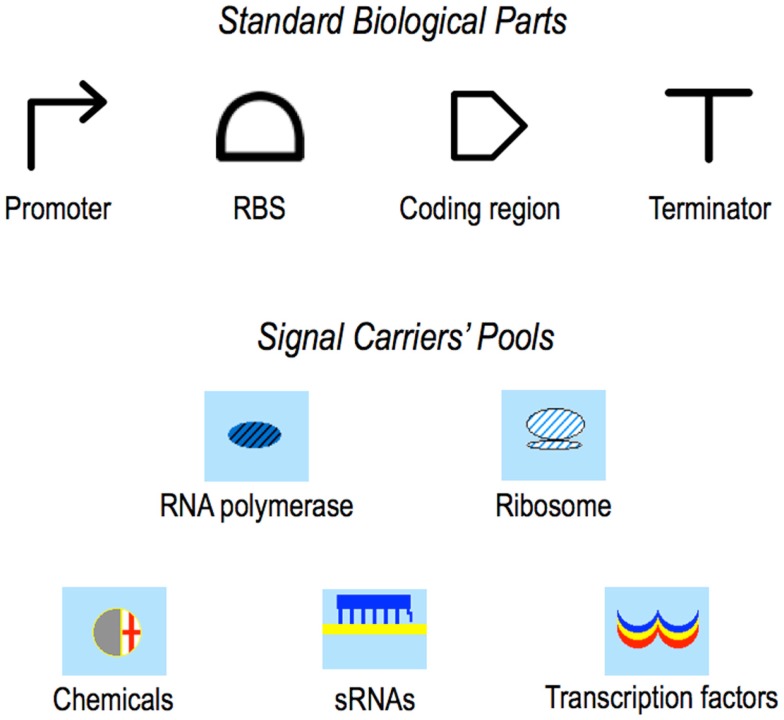
**Symbols**. In the original paper presenting Parts & Pools, Standard Biological Parts were represented by icons taken from the MIT registry. Here, the currently adopted Synthetic Biology Open Language (SBOL) (Galdzicki et al., 2014) symbols are shown. Pools’ icons did not change with respect to our first publication (Marchisio and Stelling, 2008).

In order to have both a model and a graphical representation of gene circuits where DNA traits communicate through the exchange of fluxes of signal carrier molecules, as depicted in (Endy, 2005), our method considers, beside the Standard Biological Parts, Pools of common signal carriers as basic modules for bacterial gene circuit design (see Figure 2 for the icons of bacterial Parts and Pools). Pools are abstract entities that store *free* molecules of common signal carriers. Differently from Endy’s ideas, Parts & Pools reckons as common signal carriers also transcription factors, small RNAs, and chemicals. Each of the new signal carriers is associated with a new flux: Factors Per Second (FaPS), RNAPS (RNAs Per Second), and SiPS (Signals Per Second, since chemicals are also referred to as *environmental signals*). In this way, our method merges the original picture of circuits made of transcription units exchanging activators and repressors (as in Figure 1) together with the fine-grained representation of Parts sending and receiving fluxes of RNA polymerases and ribosomes.

Pools of transcription factors and sRNAs are graphical interfaces between circuit devices, Pools of chemicals are interfaces between the whole circuit and the environment. Circuit dynamics depend on Pools’ contents that can be considered as *biological potentials*. Therefore, going on with the analogy with electronics, Pools appear to be *bio-batteries*.

**Figure 3:**
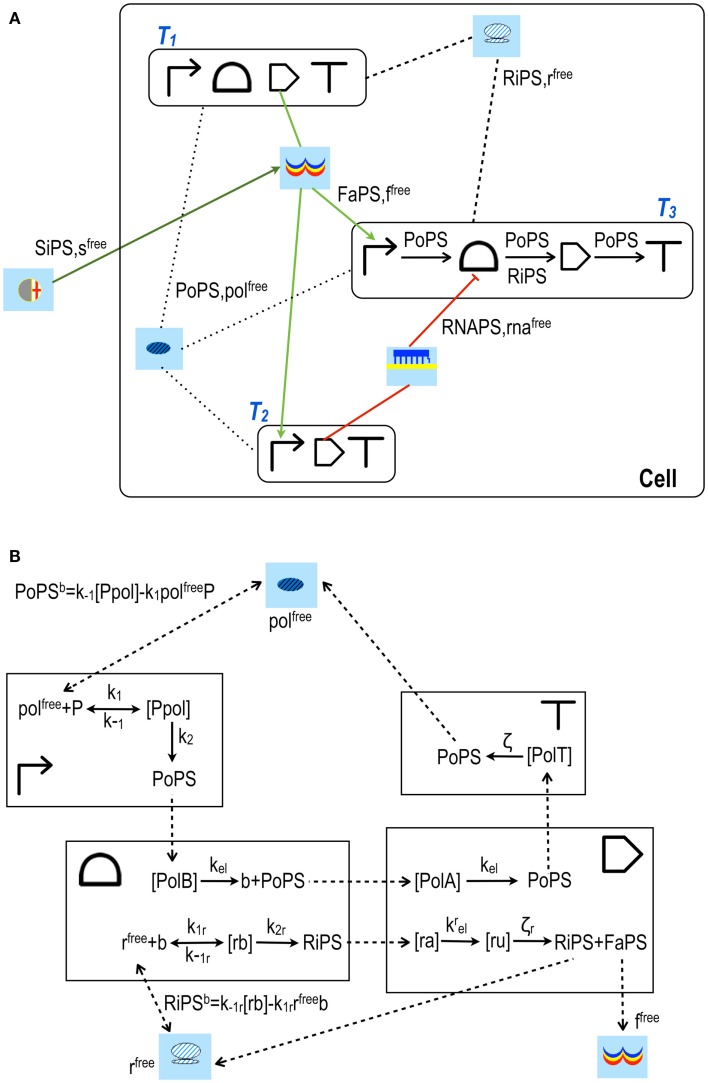
**Design and modeling in Parts & Pools framework**. **(A)** *I*1 FFL (incoherent 1 feed forward loop). The three transcription units (*T*_1_, *T*_2_, and *T*_3_) that form this network motif (Alon, 2007) are enclosed into boxes. *T*_1_ produces an activator protein that, upon binding the signal *s*, activates transcription along *T*_2_ and *T*_3_. *T*_2_ encodes for small RNAs that bind *T*_3_ mRNA at the RBS and repress the translation of the circuit readout. Parts are interested only by PoPS and RiPS fluxes, as shown inside transcription unit *T*_3_. Pools, in contrast, exchange with the connected transcription units (or Pools) both fluxes and molecules’ concentrations. **(B)** Model for transcription unit *T*_1_. RNA polymerase Pool is connected to *T*_1_ promoter and terminator, ribosome Pool is linked to *T*_1_ RBS and coding region. All the fluxes are shown as dashed arrows. PoPS*^b^* and RiPS*^b^* are bidirectional fluxes (the superscript *b* stands for *balance*) and arise from the binding/unbinding interactions between RNA polymerases (*pol^free^*) and the promoter (*P*) or between ribosomes (*r^free^*) and the mRNA (*b*) transcribed into the RBS. All the other fluxes flow in a unique direction. PoPS generated by the promoter is equal to *k*_2_[*Ppol*] and goes entirely into the species [*PolB*] belonging to the RBS Part. [*PolB*] represents a complex between RNA polymerases and the RBS DNA sequence. Similarly, PoPS flows from the RBS into the complex [*PolA*] inside the coding region (*A* refers to the *ATG* codon) and from [*PolA*] into a new complex [*PolT*] inside the terminator. Here, RNA polymerases leave the DNA and flow back (as PoPS) to their Pool. mRNA is modeled with four species: *b* and [*rb*] into the RBS, [*ra*] and [*ru*] inside the coding region. *b* is the mRNA free of ribosomes, [*rb*] represents ribosomes bound to the mRNA during the initiation phase. A RiPS flux [equal to *k*_2r_[*rb*]] is generated into the RBS and sent to the coding region where it joins the complex [*ra*] (*a* comes from the *AUG* triplet). From [*ra*], RiPS flows into a new complex [*ru*] (*u* represents the first nucleotide of a STOP codon) from which ribosomes leave the mRNA (returning, as RiPS, into their Pool) and release the activator proteins. The latter flow, as *FaPS*, into their transcription factor Pool where they become free molecules (*f^free^*) and can then interact with both *T*_2_ and *T*_3_ promoters.

Pools, however, are not just molecules’ containers but they can host bio-chemical reactions as well. Transcription factor Pools, for instance, are the place where repressors and activators form dimers or tetramers and interact with small molecules (coming from a chemical’s Pool) before binding their target promoters. Chemical Pools can host a 0th-order reaction for chemicals’ production. Moreover, in our model the total amount of RNA polymerases and ribosomes is constant whereas transcription factors, small RNAs, and chemicals are degraded into their Pools. Pools are not limited to common signal carriers but they can be associated with any other bio-chemical species in a gene circuit. We showed, for instance, how to design a bacterial pulse generating network based on an enzyme–substrate reaction (obeying a Michaelis–Menten scheme) enclosed into a dedicated Pool (Marchisio and Stelling, 2009). Furthermore, as it will be explained below, several new Pools are required to design eukaryotic gene networks. No matter which kind of species and reactions they store, Pools are connected both to Parts and other Pools in a circuit and exchange with them fluxes of molecules and species concentrations (an example of bacterial circuit is shown in Figure 3).

### Promoters and RBSs

Within the set of bacterial biological Parts the ones that require complex models are regulated promoters and RBSs. The former interact with transcription factors and RNA polymerases, the latter with ribosomes, small RNAs, and chemicals that bind structures such as riboswitches or ribozymes. Faithful theoretical representations of the promoters used in the first synthetic gene circuits in *E. coli* consider no more than only two operators. Therefore, the number of states where a promoter can lie is very small. A promoter negatively regulated by two repressors (or by a single one binding two operators) shows five different states: only one operator is *taken* by its corresponding repressor (two possible configurations); both operators are taken; both are *free* and RNA polymerase is not bound to the promoter; the two operators are free and RNA polymerase is at the promoter sequence. A symmetrical case is given by a promoter positively regulated by two activator proteins (either of the same type or of different kinds) interacting cooperatively: no activators are bound; only one is bound (two states); both are bound but RNA polymerase is not on the promoter; the operators are taken and RNA polymerase is bound to the promoter too. Slightly more complex is the case where each activator can recruit RNA polymerase independently. This scenario – used to mimic synergistic activation – demands seven states: no activator is bound; one activator is bound but RNA polymerase is not recruited (two states); one activator is bound and it has recruited RNA polymerase to the promoter (two states); both operators are taken but RNA polymerase is not on the promoter; both operators are taken and RNA polymerase is bound to the promoter as well. As for the RBS, riboswitches and ribozymes are made of one or two aptamers (Breaker, 2012) and, therefore, they bind two chemicals at most. In analogy, our framework allows no more than two sRNA binding sites. Both riboswitches/ribozymes and sRNAs regulate translation in a positive and a negative way. However, in case of small RNAs repression of translation is more common (Isaacs et al., 2006).

### Software

The software for Parts & Pools is an add-on for ProMoT (Mirschel et al., 2009). This choice is motivated by the internal language of ProMoT [Model Definition Language (MDL) (Ginkel et al., 2003)] that allows a straightforward description of modules interacting via the exchange of fluxes and molecules’ concentrations. However, as stated previously, Parts & Pools framework is conceptually independent of any specific software. Parts & Pools are first generated by running the corresponding Perl scripts. They are encoded into MDL files and modeled according to mass-action kinetics. This representation, indeed, allows a straightforward calculation of the fluxes handled by each circuit component. Parts’ and Pools’ icons are displayed on the ProMoT GUI. They contains *terminals* that are linked together via wires where information flows. In this way, gene circuits design resembles the electrical circuit design with software such as SPICE (Nagel and Pederson, 1973). Once the circuit is closed, it can be saved into a new MDL file. The bio-chemical model for the whole circuit arises from the composition of the models of its components. MDL circuit description can be exported into Systems Biology Markup Language (SBML) (Hucka et al., 2003) and Matlab (Mathworks, Nantucket/MA) format for simulations.

## Automatic Design of Bacterial Gene Digital Circuits

### Boolean gates made of DNA and mRNA

Gene Boolean gates can be engineered via promoters and RBSs regulated by one or two molecules. A single operator, aptamer, or sRNA binding site gives rise to either a YES or a NOT gate. YES gates (buffers in electronics) give 1 when their only input is present and 0 when it is absent (a NOT gate, in contrast, returns 1 in absence of its input and 0 otherwise). Parts & Pools uses models of two-operator-containing promoters to reproduce four kinds of Boolean gates: AND, NAND, OR, and NOR (see Table 2) following (Bintu et al., 2005). AND and NAND gates require cooperativity, OR gate synergistic activation. Since our model does not consider any Hill functions, cooperativity is reproduced mechanistically: the two operators are given different affinities toward their transcription factors and are referred to as strong and weak operators. When the strong operator is free, the bond between a protein and the weak operator is highly unstable whereas, when the strong operator is taken, the affinity of the weak operator toward its regulatory factor increases (higher binding and lower unbinding rate constant). Only in presence of both transcription factors is RNA polymerase recruited (AND gate) or prevented from binding the promoter (NAND gate). Cooperativity is taken into account for riboswitches/ribozymes (Mandal et al., 2004) but neglected for small RNAs. Overall, our framework mimics on the mRNA the same gates as in the transcription regulation case, a part from the OR gate. Mixed configurations (i.e., gates arising from both promoter and RBS control) give alternative designs for two-input gates (e.g., a NOR gate is achieved with a repressor together with an sRNA or an aptamer negatively regulated by a chemical) and allow the construction of 3- and 4-input Boolean gates with the only limitation that promoters and RBSs are intrinsically connected by an AND operation. These considerations brought us to characterize *in silico* a library of logic promoters and RBSs. This is a key component of our algorithm for the automatic design of gene digital circuits in bacteria (Marchisio and Stelling, 2011).

**Table 2:** **Two-input gates’ truth tables**.

a	b	AND	OR	NAND	NOR	XOR
0	0	0	0	1	1	0
0	1	0	1	1	0	1
1	0	0	1	1	0	1
1	1	1	1	0	0	0

### Digital circuits with Parts & Pools

Our algorithm borrows the Karnaugh Map method (Karnaugh, 1953) from electronics in order to translate a truth table (the only input required by the program) into two different Boolean formulas: the Conjunctive (CNF) and the Disjunctive Normal Form (DNF) formula. The former is also called Product Of Sum (POS) since it is the logic product of clauses (i.e., gates) that realize a sum (OR) of the circuit’s inputs; the latter is referred to as Sum Of Product (SOP) because, symmetrically to POS, it is a sum (OR) of clauses where circuit’s inputs are multiplied (AND). For instance, the Boolean formula corresponding to an XOR gate (see Table 2) reads in POS as $\left(a\vee b\right)\wedge \left(\bar{a}\vee \bar{b}\right),$ whereas in SOP it looks like $\left(\bar{a}\wedge b\right)\vee \left(a\wedge \bar{b}\right)$ – where ∧ means AND, ∨ OR, $\bar{a}$ stands for NOT(a), and each expression between two round brackets is a clause. In electronics, a single minimal circuit scheme corresponds to each formula: the one that minimizes the number of gates can be selected for an actual implementation. The choice of the Karnaugh Maps is justified by the fact that our algorithm deals with a small number of input signals (up to 4). Computing digital circuit schemes able to handle a larger number of inputs would require a more sophisticated algorithm such as Espresso (Brayton et al., 1984). However, such complex synthetic Boolean networks appear beyond the reach of the currently available wet-lab technology.

### Complexity score

**Figure 4:**
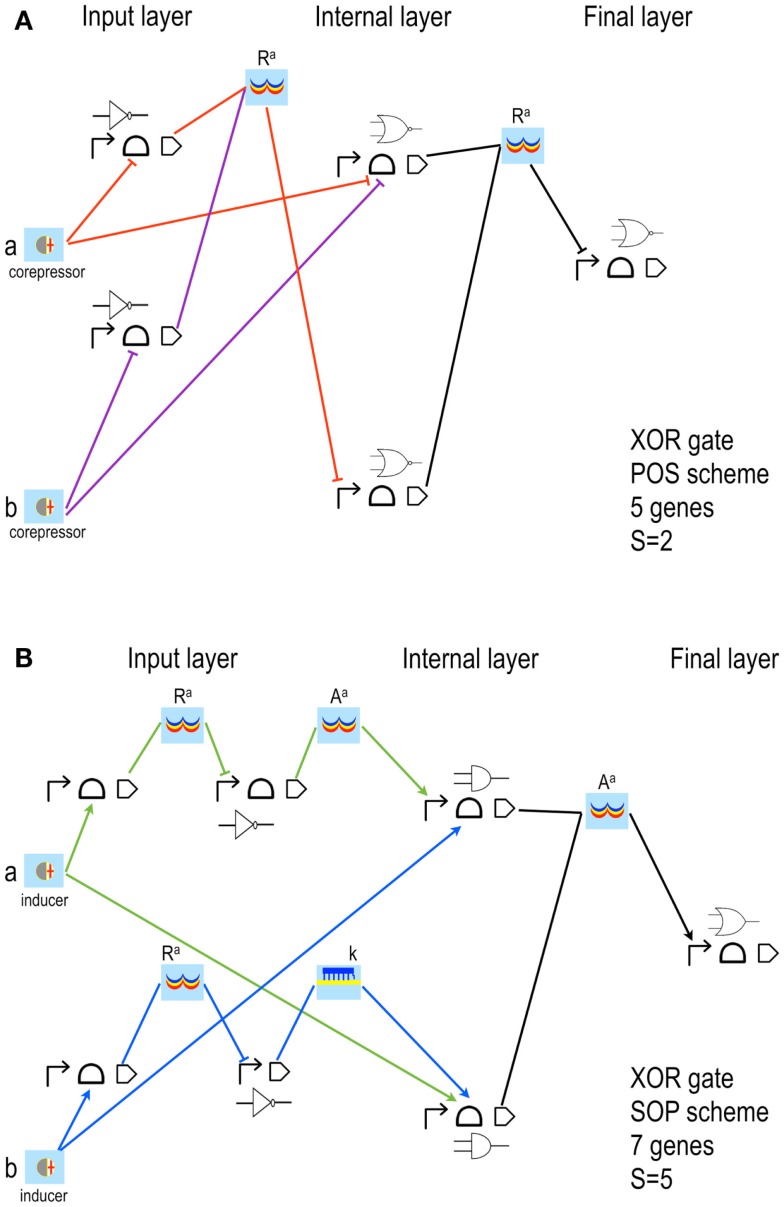
**XOR gates designed by our algorithm (Marchisio and Stelling, 2011)**. The schemes here shown have the lowest *S* both in POS **(A)** and SOP **(B)** configuration. Our algorithm is based on rather strict assumptions: POS solutions accept, as inputs, only *corepressor* chemicals able to switch off aptamers or activate repressor proteins; SOP solutions demand *inducer* chemicals that activate either aptamers or activator proteins. The input layer contains, both in POS and SOP, either YES or NOT gates. **(A)** The two corepressor inputs *a* and *b* act directly on riboswitches/ribozymes whereas their negated signals are a unique active repressor (*R^a^*). **(B)** The two inputs are inducers that activate their riboswitch/rybozyme target and their negated signals are an active activator (*A^a^*) for *a* and a *key* (*k*, an sRNA that induces translation) for *b*. Here, moreover, the two NOT gates are made of two genes. The internal layer of POS solutions is made of NOR gates (whenever a gate takes a single input, it becomes a NOT gate); AND gates (YES for a single input) are present in SOP schemes instead. NOR gates require transcription and/or translation repression; AND gates transcription/translation activation. Both circuits in figure belong to what we called *single-gate* class of solutions since their final layer is made of only one gate: NOR in POS, OR in SOP. Single-gate configurations are, generally, the least complex ones but not necessarily the most efficient. Every gate is here represented by its Parts (the terminator is always omitted) together with its electronic symbol.

In biology, several schemes per formula are possible since, differently from electronics, signal carriers are multiple. Our algorithm calculates all the possible circuit designs compatible with the two Boolean formulas. They are ranked according to the complexity score *S* that gives a possible quantification of the effort necessary for their wet-lab implementation and a criterion to select a minimal solution in biology. We define *S* as:

(1) $$S=2^{n_{R}-1}+2^{n_{A}-1}+n_{s}\;n_{R},\;n_{A}\geq 1$$

where *n_R_, n_A_*, and *n_S_* are the number of repressors, activators, and small RNAs present in a circuit scheme, respectively. This formulation is justified by the fact that most synthetic gene networks make use of the same transcription factors (e.g., LacI and TetR) and engineering new ones to avoid cross-talk among circuit components is a difficult task; small RNAs, in contrast, are easier to be constructed. Hence, circuit complexity increases only linearly in their number. Riboswitches/ribozymes do not add any complexity to the circuit scheme since chemicals act directly on them without the need for any intermediator (protein or sRNA). In each scheme designed by our algorithm, Boolean gates contain logic promoters and/or RBSs from our library and are organized in three layers named: input, internal, and final. Each layer of gates is preceded by a layer of Pools of signal carriers. Circuit inputs are chemicals, the output is a reporter protein (see Figure 4).

### Circuit performance

The main parameter to quantify gates and circuit performance is the signal separation *σ* defined as the difference between the minimal 1 (*V_m_*_1_) and the maximal 0 (*V_M_*_0_) output at steady-state:

(2) $$\sigma =V_{m1}-V_{M0}.$$

In our simulations, *σ* is expressed in protein concentration or number. With this first implementation of our algorithm, we could study in depth how circuit structure, stochastic noise, and kinetics parameter values influence circuit performance.

To assess circuit robustness, we chose eight solutions (both single-gate and disjoint ones – see below) of increasing complexity score among the 48 that our algorithm generates on the most complex truth table it can handle. Each circuit was simulated 2500 times by randomly varying, every time, the values of all its kinetic parameters (over 500) in a range of ±20% of their reference values. Having fixed in 75 proteins the minimal signal separation necessary to mimic a proper digital behavior, we found that 6 solutions overcame this threshold in at least 60% of their corresponding simulations. Moreover, a stochastic simulation performed on the least complex of the 8 circuit schemes gave *σ* approximately equal to 80 proteins.

Similarly, to estimate the incidence of leakage on the circuit performance, we varied (separately) both the transcription and translation leakage rate constants in each of our eight circuits while keeping fixed all the other parameter values. The leakage rate constants took values up to the 5% of the reference transcription or translation initiation rate constants. We obtained that only two solutions survived to the highest transcriptional leakage whereas none was able to work properly with a translation leakage rate constant ≥3% of the translation initiation rate constant.

On the basis of these results, we can claim that the Boolean networks designed by our computational tool appear to be robust to stochastic effects but sensitive to promoter and, above all, RBS leakage.

As another general result, we observe that the designs that minimize complexity have one gate only in the final layer (*single-gate* solutions, as in Figure 4). However, best performances are, in general, achieved with more entangled schemes where the final layer contains multiple gates (*disjoint* solutions). We pointed out that the signal separation of even very complex *single-gate* solution circuits can be improved by modifying only one parameter value, namely the transcription initiation rate of the promoter in the *final*-*gate* (i.e., the only gate present in the final layer) that is responsible for the output production. An experimental proof of this computational result was given recently in our work on engineering Boolean gates in budding yeast (Marchisio, 2014).

Our algorithm is the first one able to generate, in an automatic way, a collection of bacterial circuits based on fine-grained modules such as Parts and Pools. Other methods previously published (Francois and Hakim, 2004; Rodrigo et al., 2007b; Dasika and Maranas, 2008) take transcription units as basic modules and adopt less detailed bio-chemical models where, for instance, translation is treated as a single-step event. However, these methods target a broader family of gene circuits. They also need optimization algorithms, such as simulated annealing, in order to compute a circuit structure. This is not necessary in our case since the scheme of a digital circuit arises directly from its truth table and the library of logic Parts.

### Simplified algorithm

**Figure 5:**
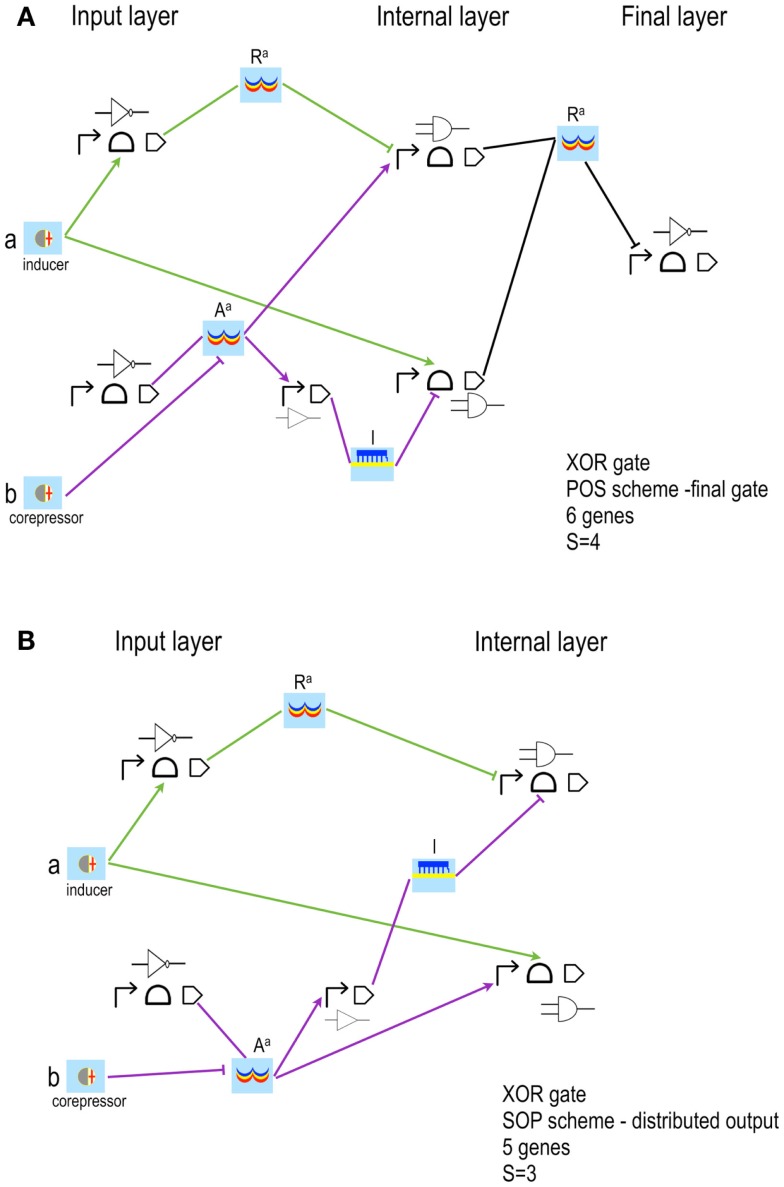
**XOR gates based on simplified designs (Marchisio and Stelling, 2014)**. The latest version of the algorithm for the automatic design of bacterial gene digital circuit allows using inducers and corepressors as inputs for both POS and SOP circuits. Furthermore, two new interactions are taken into account: inducers binding repressors and corepressors binding activators. In both cases, the chemical inactivates its target protein. The circuits here shown take as inputs the inducer *a*, which activates a riboswitch/ribozyme, and the corepressor *b*, which inhibits an activator (*A^a^*). *NOT*(*a*) is an active repressor (*R^a^*); *NOT*(*b*) a small RNA (*l*, which means *lock* since, by annealing to the mRNA, prevents ribosome binding and translation initiation). **(A)** POS solution is still organized in three layers of gates and Pools. The corresponding Boolean formula has been rearranged as: $NOT\left(\left(\bar{a}\wedge \bar{b}\right)\vee \left(a\wedge b\right)\right).$ **(B)** SOP solution is designed according to the distributed output architecture. This implies a reduction in the complexity score with respect to the corresponding POS configuration.

As a drawback, the majority of the digital circuits designed by our software are too complex for a wet-lab implementation. Following the work by Regot et al. (2011), we came up with a new algorithm where circuit schemes are simplified (Marchisio and Stelling, 2014). Together with the truth table, the software requires, as inputs, the kind of the chemicals to be sensed (i.e., inducers or corepressors) and if they bind a repressor, an activator, or a riboswitch/ribozyme. Moreover, only two or three input chemicals are taken into account. In this way, the number of solutions per Boolean function is considerably reduced. The input is translated into CNF and DNF formulas by means of the Karnaugh Map method again. However, circuits in POS and SOP are now based on the same kinds of basic Boolean gates: YES, NOT, and AND. Notice that here with AND we mean “multiplicative” gates i.e., each of their inputs can be either positive or negative (to be more precise, a two-input gate obeying the expression $\left(\bar{a}\wedge b\right)$ should be referred to as N-IMPLY, for instance). As a consequence, we modified our library of logic Parts with the addition of two-operator-containing promoters that are bound by one repressor and one activator, and two-aptamer-containing RBSs, one activated and the other repressed by different chemicals. Moreover, our new algorithm considers only small RNAs that repress translation. This is their most common function, and it can be easily extended to RNA interference in eukaryotic gene circuits. Circuits in SOP are designed according to the *distributed output architecture*. Here, the circuit output is produced by each AND gate in the internal layer and the final gate is no longer necessary. This structural simplification permits our method to save one gene and the corresponding regulatory factor. POS configurations want a final NOT gate always (see Figure 5). Even though they are, in general, more complex than the corresponding SOP they might be chosen for a wet-lab implementation in order to increase the circuit performance that, as we have seen above, often requires higher structural complexity. Finally, when the input chemicals do not bind any riboswitch/ribozyme, a solution based only on transcription regulation is always computed. Despite its high complexity score, it might be preferred to the ones that mix repressors and activators with sRNAs since transcription factors are still more exploited than any translation regulation mechanism.

## Eukaryotic Modules

### Rule-based modeling

In order to prove that our first computational tool could provide valuable alternative designs to synthetic gene digital circuits in literature, we chose as a benchmark the *RNAi-based logic evaluator* (Rinaudo et al., 2007). This circuit computes a rather complex Boolean formula: $\left(a\wedge b\wedge d\right)\vee \left(\bar{a}\wedge c\right)$ by using five small interfering RNAs, one for each signal (positive or negative). The OR gate is replaced by a distributed output configuration since both AND gates produce the circuit readout (fluorescence). By considering siRNAs as analogous to bacterial small RNAs, we assign to this circuit a complexity score *S* = *5*. Our algorithm is able to produce 15 solutions with a lower complexity score. The least complex solution requires two repressors and four riboswitches for five genes overall arranged in a POS scheme. However, since no eukaryotic module was present in our tool, we could not compare the performance *in silico* of our solution and the original circuit. Rinaudo and co-authors network demands Pools for RNAi and mRNA maturation, cell compartments, and a model for translation regulated by more than two RNA molecules. In general, bio-chemical descriptions of eukaryotic promoters and mRNAs that are regulated by several factors can be affected by a combinatorial explosion of the number of the states they lie in. A common technique to cope with a typically exponential number of species and reactions is *rule-based* modeling. In this context, a system is specified by giving an abstract representation of the species involved, general rules explaining the way they interact, a list of the species present at the beginning of the system simulation together with their concentrations, and the kinetic parameter values associated with the reaction rules. On this input, software such as BioNetGen (Blinov et al., 2004) and Kappa (Danos and Laneve, 2004) give a detailed system description in terms of species and reactions.

**Figure 6:**
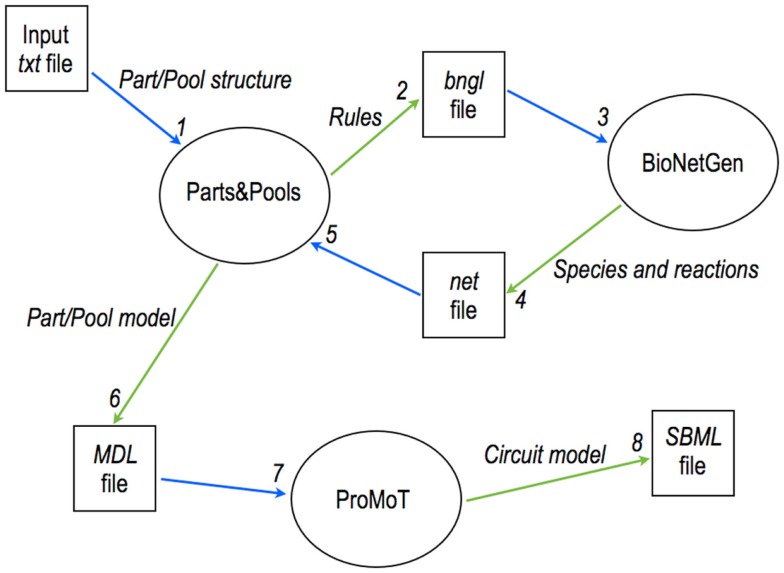
**Modular, rule-based modeling with Parts & Pools**. A text file is converted into an MDL file containing a eukaryotic module description via the interaction of Parts & Pools and BioNetGen. MDL files are loaded into ProMoT where gene circuits are designed and exported, for instance, into SBML format for simulations and analysis.

Parts & Pools employs BioNetGen to compute models for eukaryotic circuit components (Marchisio et al., 2013). However, BioNetGen generates *per se* a faithful representation of a closed biological system but not of open modules that should be wired together into bigger systems such as synthetic gene circuits. More precisely, BioNetGen cannot compute the module interface i.e., the fluxes and the concentrations of signal carriers – and other molecules – that a module exchanges inside a circuit. Therefore, Parts & Pools was extended to the eukaryotic cells by merging the high modularity offered by the MDL together with BioNetGen’s rule-based modeling. BioNetGen is required only for complex Parts, such as regulated promoters, and complex Pools, such as the ones containing regulated mature mRNA. All the other circuit components do not demand a rule-based modeling approach. Eukaryotic Parts & Pools is a collection of Python and Perl scripts. Together with the ones that generate the files of Parts and Pools, there are three more scripts that return models for the compartments (nucleus and cytoplasm) and the whole cell. Scripts for Parts and Pools process an input text file where the module structure and interactions are described (e.g., for a promoter: number and kind of transcription factors, number of operators per transcription factors, cooperativity, and interaction with chemicals) and parameter values are specified. This information is translated into instructions (rules) in the BioNetGen Language (BNGL). BioNetGen is called and provides, on the basis of these rules, a list of species and reactions present in the module. This is sent back to our script that calculates the fluxes and molecules concentrations handled by the module and writes an MDL file that contains a complete module description made of its interface together with its species and reactions (see Figure 6).

### Complex bio-components and gene circuits

**Figure 7:**
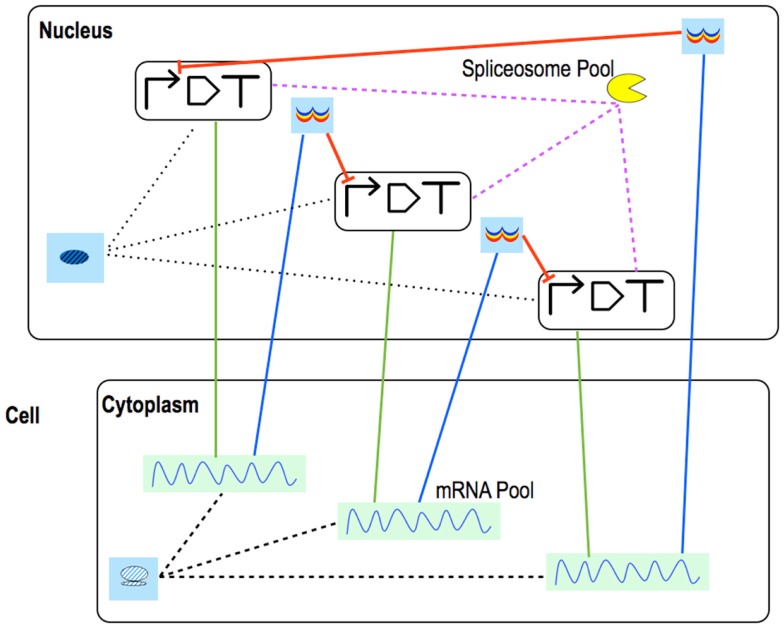
**Eukaryotic repressilator**. The repressilator scheme is drawn with eukaryotic Parts & Pools. Green straight lines represent transcription, blue ones, translation. mRNA maturation is modeled into each coding region where pre-mature mRNA interacts with the spliceosome. This requires a link between each transcription unit and the spliceosome Pool. Mature mRNA is exported into a Pool in the cytoplasm where translation takes place. Once synthesized, repressors are brought to their Pools in the nucleus from where they can flow to their target promoters and inhibit transcription.

In our representation, eukaryotic promoters can be regulated by *N_t_* transcription factors, each binding to *N_o_* operators. Cooperativity is allowed only between proteins of the same species i.e., hetero-cooperativity is no longer taken into account. Activators can recruit RNA Polymerase independently of each other without implying synergistic activation (as it was in the bacterial case). The ribosome binding site does not exist as an independent Part in eukaryotes: translation regulation takes place into the mature mRNA Pools. Each mRNA chain can host *N_r_* riboswitches/ribozymes. They have either one or two aptamers. Tandem riboswitches show both homo- and hetero-cooperativity (Jose et al., 2001; Mandal et al., 2004). Moreover, *N_s_* siRNAs can bind the mRNA, each one to *N_b_* different binding sites. As soon as an siRNA (bound to the RISC–RNA induced silencing complex) anneals to its target sequence, the mRNA is degraded. Differently from the bacterial framework, the mRNA half life is here determined by a terminator (Yamanishi et al., 2011). Pools are used to model new reactions. RNA interference requires placing a Pool for the Dicer enzyme in the nucleus and a Pool for the RISC complex in the cytoplasm; mRNA maturation and splicing demands a Pool for the Spliceosome in the Nucleus (a eukaryotic circuit scheme is shown in Figure 7).

With the new set of eukaryotic Parts and Pools we are able to reconstruct the original circuit by Rinaudo and co-authors. It consists of 12 genes for 197 species and 474 reactions. Simulation results are in a fair agreement with the published data. Importantly, we did not have to run an optimization algorithm and fine-tune parameter values in order to achieve a good match between computational and experimental data but we made use only of parameter values taken or derived from previous works. In order to test our algorithm on even more complex networks, we designed a transcriptional counterpart of the RNAi-based logic evaluator. This circuit gathers 7 genes for a total of 187 species and 1165 reactions. Circuit simulations reproduce the circuit truth table faithfully pointing out that our modeling framework for eukaryotic modules – the first in Synthetic Biology – is highly reliable and might be adopted to study *in silico* future novel eukaryotic gene circuits.

## Discussion

Parts & Pools is a framework for the modular design and modeling of synthetic gene circuits both in bacterial and eukaryotic cells. Bacterial Parts & Pools was used to model Boolean gates and led to the development of an algorithm for the automatic design of gene digital circuits.

The software implementing Parts & Pools is an add-on of ProMoT whose internal language, MDL, makes it straightforward to define modules that communicate via the exchange of fluxes and concentrations of molecules. Eukaryotic modules, which can host a combinatorial number of species and reactions, require a rule-based modeling approach. Therefore, scripts of Parts & Pools that generate a formal description for complex eukaryotic circuit components (such as promoters and mRNA Pools) call the software BioNetGen to get a complete list of the species and reactions wrapped by these modules.

Recently, Parts & Pools was used to drive the construction of small transcriptional networks in yeast (Marchisio, 2014). In particular, a design strategy to improve Boolean gate and circuit signal separation – pinpointed by our algorithm for the automatic gene digital circuit design – was used and proved to be valid by re-engineering a YES and an AND gate in *S. cerevisiae*. In that work, we managed to mimic an increase in the strength of the final-gate promoter (as suggested by our tool) via a double integration in the yeast genome (Ajo-Franklin et al., 2007; Blount et al., 2012).

Parts & Pools can be adopted to design, simulate, and help the wet-lab implementation of a large number of synthetic gene circuits. We want to stress that fluxes of signal carriers such as PoPS and RiPS are useful interfaces for modular gene circuit design *in silico* but their knowledge *in vivo* is not necessary to check Parts & Pools predictions on circuit behavior. Indeed, as shown in (Marchisio, 2014), computed steady-state concentrations of the circuit protein output can be easily compared to experimental data (fluorescence levels) after rescaling them to given reference values.

What is still missing in Parts & Pools, in order to support wet-lab circuit construction, is a clear correspondence between model Parts and DNA sequences. This will require to establish connections to existing or new databases, to handle biological components annotated in Synthetic Biology Open Language (SBOL) format (Galdzicki et al., 2014), and to consider alternative models to the current one fully based on mass-action kinetics. Indeed, the theoretical representation of Parts & Pools is, at present, very detailed and demands the knowledge of parameter values that are still too difficult to be measured in wet-lab experiments.

As a further future improvement, the automation process of gene digital circuit design should be enabled to produce not only abstract schemes but also actual gene gates. This might be achieved through a Directed Acyclic Graph (DAG)-based approach [as in iBioSim (Roehner and Myers, 2014)] by using the signal separation in Eq. (2) as a cost function to be optimized.

## Conflict of Interest Statement

The author declares that the research was conducted in the absence of any commercial or financial relationships that could be construed as a potential conflict of interest.
